# Effect of orthodontic treatment on the periapical radiolucency of endodontically treated teeth: a CBCT analysis

**DOI:** 10.1186/s12903-023-02907-1

**Published:** 2023-05-13

**Authors:** Sunhong Kim, Soo-Jeong Hwang, Min-Seock Seo

**Affiliations:** 1 Department of Conservative Dentistry, Wonkwang University Daejeon Dental Hospital, 77 Dunsan-Ro, Seo-Gu, 35233 Daejeon, Republic of Korea; 2 Department of Dentistry, Doonsan Health Promotion Center, Daejeon, Korea

**Keywords:** Periapical radiolucency, Cone-beam computed tomography, Root canal treatment, Orthodontic treatment

## Abstract

**Background:**

This study aimed to evaluate periapical radiolucency of endodontically treated teeth before and after orthodontic treatment using cone-beam computed tomography (CBCT).

**Methods:**

Patients who underwent orthodontic treatment at Wonkwang University Daejeon Dental Hospital between January 2009 and June 2022 were included based on the following criteria: root canal treatment, and availability of CBCT images taken before and after orthodontic treatment with an interval of > 1 year between both scans. Patients with primary teeth or orthodontic tooth extractions were excluded. The size of the periapical radiolucency (SPR) of the endodontically treated tooth was evaluated using CBCT. Pre-orthodontic treatment CBCT images and the latest post-orthodontic treatment CBCT images were analyzed. The selected teeth were further categorized based on the orthodontic duration, CBCT interval, the patient sex and age, the tooth type and position (maxilla or mandible), and quality of root canal obturation. Statistical analyses were performed to evaluate changes in SPR using the paired t-test and multiple regression analysis.

**Results:**

In total, 115 teeth (37 anterior teeth, 22 premolars and 56 molars) from 61 patients (age, 14–54 years) were included, with 39 teeth from male patients and 76 teeth from female patients. The age was ranged between 14 and 54 years old, and mean age was 25.87 years old. The mean CBCT interval and orthodontic treatment period were 43.32 months and 36.84 months, respectively. Seventy-five teeth showed good obturation quality, 80 were not used as anchors during orthodontic treatment, and 71 were maxillary. The SPR size increased after orthodontic treatment for 56 teeth and decreased for 59 cases. The average change in SPR was -0.102 mm and the difference was not significant. Significant decrease of SPR were observed between female patients (*p* = 0.036) and maxillary teeth (*p* = 0.040).

**Conclusion:**

Orthodontic treatment had no significant impact on the changes in the SPR in endodontically treated teeth in most categories. However, there was a significant difference among females and the maxillary teeth. In both categories, the size of radiolucency decreased significantly.

## Background

Endodontic treatment is aimed at preventing apical periodontitis and establishing favorable conditions for the recovery of periapical tissues. Since apical periodontitis is an infectious condition, the goal of endodontic treatment is to eliminate infection. Microorganisms are removed through appropriate mechanical and chemical disinfection. Elimination of irritants and root canal obturation reduce inflammation and promote healing of the periapical lesion [1]. This further induces the bone regeneration in the damaged periapical area. Bone regeneration is indicative of periapical changes and presents as increased opacity on radiographs. Thus, reduction in periapical radiolucency after successful endodontic treatment can be confirmed radiographically [2, 3].

Rearrangement of teeth through orthodontic movement involves application of forces to the periapical region, induces resorption and remodeling of alveolar bone. Resorption of alveolar bone appears as an inflammatory response, similar to that caused by osteoclastic activity in inflammatory reactions. The resorption occurs in the direction in which the force of orthodontic treatment is applied [4, 5].

Currently, many patients undergo orthodontic treatment for aesthetic benefits. Orthodontic treatment may affect the pre-existing lesions in patients who have undergone endodontic treatment. In fact, orthodontic treatment can lead to root resorption, which proceeds similarly to bone resorption due to the inflammatory reaction in the periapical lesion [6]. Due to this inflammatory reaction, orthodontic treatment may cause alterations in pre-existing periapical lesions in addition to root resorption. No previous studies have investigated the effect of applying orthodontic force on the periapical radiolucency of endodontically treated teeth.

The purpose of this study was to evaluate periapical radiolucency around endodontically treated teeth before and after orthodontic treatment using cone-beam computed tomography (CBCT). The null hypothesis of the study was that the size of periapical radiolucency from endodontically treated teeth changes with orthodontic treatment.

## Methods

### Ethical statements

This study was approved by the Institutional Review Board of Daejeon Dental Hospital, Wonkwang University College of Dentistry (approval no. W2205/001–001).

### Patients’ selection

Patients who had been treated between January 2009 and June 2022 in the Department of Orthodontics, Wonkwang University Daejeon Dental Hospital were investigated.

For inclusion criteria, patients who had undergone CBCT for pre-treatment evaluation before orthodontic treatment were selected. Of them, patients who underwent orthodontic treatment and had subsequent CBCT images taken at least one year after the first CBCT scan were enrolled. If patients undergone multiple CBCT scans, the latest images were used for evaluation. Patients with teeth that had a previous history of endodontic treatment were selected and their CBCT images were analyzed. The size of the periapical radiolucency (SPR) of the endodontically treated tooth (ETT) was evaluated using CBCT.

Teeth that were extracted during orthodontic treatment and primary teeth were excluded from the study. CBCT scans were performed using CS 8100 3D ® device (Carestream, Rochester, New York, USA), operating at 90 kV and 3.2 mA, 18 s of exposure time with a voxel dimension of 150 µm and generating DICOM format images. The selected teeth were further categorized based on the orthodontic treatment duration, CBCT interval, patient sex and age, type of teeth, and position of teeth (maxilla or mandible).

### Measuring the size of periapical radiolucency

The largest linear distance of the radiolucency was measured by evaluating the axial, sagittal, and coronal planes of the CBCT images, and this length was selected as the SPR. SPR was measured on CBCT images using INFINITT PACs software (INFINITT Healthcare, Seoul, South Korea) and On3D software (3D ONS, INC., Seoul, South Korea). The SPR of less than 0.5 mm was considered periodontal space and was therefore excluded from the analysis [7].

To evaluate the changes in SPR, CBCT images obtained at two different time points were evaluated: 1) pre-treatment CBCT images, before orthodontic treatment (BO CBCT) and 2) latest CBCT images, after orthodontic treatment (AO CBCT). SPR was measured by one operator, twice at 2-week intervals, and manual adjustment was performed. This method was as implemented in a previous study by Liang et al. [8], who measured the SPR on CBCT images at 2-week intervals and reproducible results were obtained (intraclass correlation coefficient [ICC] = 0.991).

### Evaluation of the quality of root canal obturation

BO CBCT images were analyzed to evaluate the quality of root canal obturation using two standards:- 1) distance from the apical end of the root to the canal obturation materials and 2) presence of voids in the obturation material. The obturation quality was classified into three categories:- 1) Good, 2) Moderate, and 3) Poor. The classification criteria used in this study are different from those used previously [9, 10] due to the addition of a “no canal obturation” category. The classification criteria were as follows:

- 1) Good: when the distance from the periapical end to the obturation material is 0–2 mm with no voids in the obturation material.
- 2) Moderate: the distance is within 0–2 mm, but voids in the obturation material are detected on CBCT images or the obturation material is unevenly distributed.
- 3) Poor: the distance is 2 mm or more, in case of overfilling, and in cases with missed canals in multi-root tooth.
- 4) No canal obturation: conventional access opening and removal of pulp chambers but root canals remain intact, with no obturation material in any canal.

Through CBCT imaging using On3D software (3D ONS, Inc.), the distance from the apical end of the root to the canal obturation materials was measured along with the evaluation of the SPR, and the presence of voids in the obturation material was investigated.

### Identifying teeth used as anchors in orthodontic treatment

A tooth can be used as an anchor to maintain space after extraction of adjacent teeth or to retract an impacted tooth during orthodontic treatment. Whether the ETT was used as an anchor was analyzed using medical records from the orthodontic department. First, it was determined if the teeth were used as a fixation source, and if this induced changes in the SPR were analyzed using CBCT images from BO and AO CBCT.

### Statistical analysis

SPSS Statistics software (version 16.0; SPSS, Inc., Chicago, IL, USA) was used for statistical analysis. The parameters followed normal distribution (*p* > 0.05). A paired t-test was used to analyze SPR. BO and AO CBCT images were compared, and the changes in SPR diameter changed was evaluated using a paired t-test. Multiple regression analysis was performed to determine whether there was a significant difference between the classified categories. Statistical significance was set at *p* < 0.05.

## Results

A total of 115 teeth from 61 patients were evaluated: 76 teeth were from female patients (66.1%), 56 molars (48.7%), and 74 were maxillary (61.7%). The age was ranged between 14 and 54 years old, and mean age was 25.87 years old. Most teeth were classified as having good obturation quality (65.3%). Further, most teeth were not used as anchors (69.6%). The detailed findings are presented in Table 1.

**Table 1:** Demographic characteristics and clinical data of total variables

Variables	Case number	Percentage
**Demography**		
***Sex***		
Male	39	33.9%
Female	76	66.1%
***Age***		
10—19	23	20%
20 – 29	63	54.8%
30 – 39	21	18.3%
$\ge$ 40	8	7.9%
***Orthodontic treatment period (Months)***		
0 – 24	26	22.6%
24 – 36	41	35.7%
36 – 48	18	15.6%
$\ge$ 48 0ㅇ -	30	26.1%
***CBCT interval (Months)***		
12 – 24	19	16.5%
24 – 36	636	31.3%
36 – 48	26	22.6%
48 – 60	9	7.8%
60 – 72	10	8.7%
$\ge$ 72	15	13.1%
***Type of tooth***		
Anterior	37	32.2%
Premolar	22	19.1%
Molar	56	48.7%
***Position of tooth***		
Maxillary	71	61.7%
Mandibular	44	38.3%
***Canal obturation quality***		
Good	75	65.3%
Moderate	18	15.7%
Poor	18	15.7%
No canal obturation	4	3.5%
***Whether used as an anchor***		
Yes	30	26.1%
No	80	69.6%
Unknown	5	4.3%

The SPR had increased for 56 teeth and had decreased for 59 teeth. The change in the SPR of all teeth was not significant (Table 2).

**Table 2:** Paired t-test results of the changes in the SPR in 115 teeth (average [mm] and SD)

		n	Percentage (%)	Average SPL BO CBCT (mm)	Average SPL AO CBCT (mm)	SPL change Average(mm) $\pm$ SD	*p*—value
**Changes of SPL**	Total	115		2.52	2.41	-0.102 $\pm$ 1.13	0.315
	Lesion Decreased	59	51.3%	2.80	1.98	-0.817 $\pm$ 0.97	0.000
	Lesion Increased	56	48.7%	2.22	2.86	0.642 $\pm$ 0.75	0.000

*SPL* Size of the periapical lesion

*CBCT* Cone-beam computed tomography

*BO CBCT* CBCT images before orthodontic treatment

*AO CBCT* CBCT images after orthodontic treatment

Paired-t test was used to evaluate whether the difference in SPR change was significant. The p -values are summarized in Table 3. There was a significant difference among females (*p* = 0.036). The maxillary teeth also showed a significant difference (*p* = 0.040). Multiple regression analysis was conducted to determine whether there was a significant difference between the categories. A standard method of simultaneous entry was used. All the independent variables were entered into the equation simultaneously. The details and *p* -values are summarized in Table 4. In the multiple regression analysis, teeth of female patients showed a significant decrease in sizes of periapical radiolucency (*p* = 0.041). The ICC value for intra-observer agreement or the rating scores 0.953, indicating that this method is reproducible.

**Table 3:** Paired t-test analysis results of SPR measurement

Variables	BO CBCT	AO CBCT	Changed	*p*—value
	Average(mm) $\pm$ SD	Average(mm) $\pm$ SD	Average(mm)	
***Sex***				
Male (*n* = 39)	2.40 $\pm$ 1.39	2.62 $\pm$ 1.57	0.22	0.218
Female (*n* = 76)	2.57 $\pm$ 1.55	2.30 $\pm$ 1.28	-0.27	**0.036**
***Age***				
10 – 19 (*n* = 23)	3.01 $\pm$ 2.03	2.66 $\pm$ 1.73	-0.35	0.264
20 – 29 (*n* = 63)	2.44 $\pm$ 1.38	2.28 $\pm$ 1.27	-0.16	0.251
30 – 39 (*n* = 21)	2.03 $\pm$ 0.84	2.23 $\pm$ 1.21	0.20	0.262
$\ge$ 40 (*n* = 8)	2.94 $\pm$ 1.44	3.20 $\pm$ 1.48	0.25	0.203
***Orthodontic treatment period (Months)***				
0 – 24 (*n* = 26)	2.54 $\pm$ 1.58	2.37 $\pm$ 1.26	-0.16	0.430
24 – 36 (*n* = 41)	2.76 $\pm$ 1.61	2.58 $\pm$ 1.45	-0.18	0.211
36 – 48 (*n* = 18)	1.93 $\pm$ 0.81	2.24 $\pm$ 1.52	0.31	0.210
$\ge$ 48- (*n* = 30)	2.52 $\pm$ 1.52	2.31 $\pm$ 1.36	-0.21	0.451
***CBCT interval (Months)***				
12 – 24 (*n* = 19)	2.49 $\pm$ 1.57	2.59 $\pm$ 1.38	0.10	0.575
24 – 36 (*n* = 36)	2.67 $\pm$ 1.71	2.47 $\pm$ 1.50	-0.20	0.160
36 – 48 (n = 26)	2.43 $\pm$ 1.36	2.08 $\pm$ 1.45	-0.34	0.205
48 – 60 (*n* = 9)	2.06 $\pm$ 1.03	2.70 $\pm$ 1.49	0.64	0.112
60 – 72 (*n* = 10)	2.03 $\pm$ 0.86	1.97 $\pm$ 0.89	-0.07	0.850
$\ge$ 72 (*n* = 15)	2.94 $\pm$ 1.63	2.74 $\pm$ 1.20	-0.21	0.639
***Type of tooth***				
Anterior (*n* = 37)	2.55 $\pm$ 1.20	2.44 $\pm$ 1.35	-0.10	0.592
Premolar (*n* = 11)	2.22 $\pm$ 1.24	1.89 $\pm$ 1.76	-0.33	0.178
Molar (*n* = 56)	2.61 $\pm$ 1.76	2.60 $\pm$ 1.53	-0.02	0.901
***Position of tooth***				
Maxillary (*n* = 71)	2.54 $\pm$ 1.43	2.29 $\pm$ 1.33	-0.25	**0.040**
Mandibular (*n* = 44)	2.47 $\pm$ 1.60	2.60 $\pm$ 1.46	0.13	0.504
***Canal obturation quality***				
Good (*n* = 75)	2.59 $\pm$ 1.71	2.50 $\pm$ 1.53	-0.08	0.561
Moderate (*n* = 18)	2.58 $\pm$ 1.16	2.40 $\pm$ 1.56	-0.18	0.459
Poor (*n* = 18)	2.32 $\pm$ 0.73	2.32 $\pm$ 1.35	-0.00	0.995
No canal obturation (*n* = 4)	2.03 $\pm$ 0.85	2.05 $\pm$ 1.07	0.02	0.889
***Whether used as an anchor***				
Yes (*n* = 30)	2.37 $\pm$ 1.31	2.26 $\pm$ 1.18	-0.11	0.584
No (*n* = 80)	2.58 $\pm$ 1.59	2.44 $\pm$ 1.46	-0.15	0.251
Unknown (*n* = 5)	2.27 $\pm$ 0.75	2.83 $\pm$ 1.48	0.56	0.418

*SPL* Size of the periapical lesion

*CBCT* Cone-beam computed tomography

*BO CBCT* CBCT images before orthodontic treatment

*AO CBCT* CBCT images after orthodontic treatment

^*^ Paired t-test / Bold and underline values indicate statistically significant differences (*p* < 0.05)

**Table 4:** Multiple regression analysis results of SPR changes

Variables		Unstandardized Coefficients		Standardized Coefficients	t	*p* – value
		**B**	**Standard error**	**β**		
Constant		1.272	0.882		1.442	0.152
Sex (Ref: male)		-0.525	0.238	-0.213	-2.205	**0.041**
Age		-0.02	0.016	-0.012	-0.108	0.914
Orthodontic period		-0.037	0.091	-0.050	-0.402	0.688
CBCT interval		0.016	0.061	0.029	0.254	0.800
Type of tooth (Ref: Incisor)	Premolar	-0.016	0.185	-0.012	-0.085	0.932
	Molar	0.013	0.010	0.133	1.385	0.169
Position of tooth (Ref: maxilla)		-0.539	0.341	-0.225	-1.582	0.117
Obturation quality		-0.005	0.127	-0.04	-0.041	0.968

Bold and underline values indicate statistically significant differences between groups (*p* < 0.05)

*SPL* Size of the periapical lesion

$${R}^{2}=0.131,$$ Adjusted $${R}^{2}=0.066$$, $$F-value= 2.001$$ (*p* < 0.05)

## Discussion

Root resorption is a common adverse effect of orthodontic treatment [4, 6] and has been studied extensively. Root resorption occurs more prominently in teeth receiving a strong orthodontic force or those undergoing extensive movement through orthodontic treatment [4]. Root resorption initiates with orthodontic forces and proceeds similar to bone resorption due to inflammation in the periapical lesion.

The magnitude and duration of the orthodontic force could be analyzed based on whether the tooth was used as an anchor during orthodontic treatment and the duration of orthodontic treatment. Anchor teeth may receive more intense force during orthodontic treatment, hereby affecting periapical lesions. Orthodontic forces applied to each tooth were analyzed and used as anchors during orthodontic treatment were identified. As the orthodontic treatment period increases, the tooth may experience prolonged orthodontic forces.

To our knowledge, there are no prior studies on the effect of orthodontic treatment on an ETT and its periapical radiolucency. Therefore, this study is different from previous studies in that we evaluated the periapical radiolucency of teeth with previous endodontic treatment history using CBCT.

In this study, there was no clear correlation between changes in SPR of ETT and orthodontic treatment. The effect of orthodontic forces on periapical radiolucency at the ETT was not evident in this study. The average change in SPR was -0.102 mm, and no significant difference was noted *(p* > *0.05)*. In a histological study of periapical lesions and orthodontic forces, Mah et al. [10] reported no significant difference. Although there was a discrepancy in the sample selection process, there was no correlation between orthodontic treatment and changes in existing periapical radiolucency.

The difference in the change in SPR was insignificant in most categories, which led to partial rejection of the null hypothesis that orthodontic treatment affects changes in periapical radiolucency.

On analyzing the factors that could potentially affect SPR, patient ages, type of tooth, and quality of endodontic treatment showed no significant effects. However, radiolucency in female patients showed significant changes in SPR. A decrease in SPR was evident in the female patients. In a previous study [11] evaluating root resorption, male patients showed more evident resorption than female patients. This may be explained by hormonal changes affecting inflammatory responses in woman. Amaro et al. [12] studied the teeth and periodontal tissues of rat and found that root resorption is more obvious in case of insufficient estrogen secretion. Estrogen plays an important role in homeostasis in the alveolar bone [13, 14] and may have been associated with a significant reduction in the SPR in female patients.

Compared to mandibular teeth, maxillary teeth exhibited a significant difference in the change in SPR, which dramatically decreased after orthodontic treatment. In a study by Hajihassani et al. [15], endodontically treated mandibular molars had significantly larger apical lesions than maxillary molars, and mandibular molars showed larger lesions in the furcation area. Buccal and lingual cortical bone of the mandible may resist the spread of infection, causing the lesions to expand in the vertical direction than the horizontal. Further, the presence of the maxillary sinus may affect SPR. The presence of sinuses reduces bone volume and size, inevitably decreasing SPR. These differences between the maxilla and mandible may contribute to the significant changes in SPR after orthodontic treatment.

The quality of root canal obturation can be assessed based on the length from the obturation material to the root apex. According to previous studies [16–18], obturation quality affects the prognosis of teeth that have undergone endodontic treatment and have periapical lesions. Teeth with obturation materials shorter than 2 mm from the apex showed a higher healing rate during apical surgery [19–21]. In the study by Eckerbom et al. [22], better quality of endodontic treatment showed the reduction of SPR more clearly through healing of the periapical lesions. However, in this study, radiolucency changes showed no significant differences according to the quality of the root canal obturation. Most teeth were classified as having good obturation quality, with 75 of 115 teeth (65.2%). There were 18 teeth each classified as having moderate and poor quality obturation, and only 4 teeth had no canal obturation.

Additionally, it was confirmed that the longer the follow-up period, the greater was the healing of the radiolucency. The mean CBCT interval in this study was 36 months. In this study, the CBCT intervals of the patients represent the patients’ follow-up period. The longest follow-up period was 8 years and 10 months, and the average follow-up period was 36 months. In most cases, the CBCT interval was less than 4 years, and 25 cases had follow-ups more than 5 years. Although a 36-month follow-up is not short, a longer duration would have been preferable.

Patient age also did not significantly affect SPR. In other studies evaluating changes in lesions using CBCT after apical surgery, the effects of patient age on lesion healing were different. According to Pallares-Serrano et al. [23], patient age of 20 years or younger had a significant effect on the healing of the lesion, but Liao et al. [21] showed that there was no significant effect of age on healing. Among the teeth and radiolucency analyzed in this study, 21 were of patients in their 30 s, 8 of patients in their 40 s and older, and most teeth were of patients in their teens and twenties. For teeth of patients in their 30 s and 40 s, the proportion of the group in which the SPR increased after orthodontic treatment was 42.9% and 50%, respectively, which was higher than the 31.8% among patients in their 20 s, but there was no significant difference. As the proportion of patients aged more than 30 years was significantly smaller, this insignificant difference may also be attributed to discrepancies in the samples.

CBCT was used to analyze the SPR. CBCT images are more accurate than periapical and panoramic radiographs in detecting and measuring the periapical lesions [24]. Usually, periapical lesions are evaluated on CBCT images using periapical indices, such as PAI and CBCTPAI [24, 25]. Periapical lesions were presented with scores according to the size, location, and/or level of bone damage in these cases. When used to assess the existence of periapical lesions and apical periodontitis, scoring with the index produced accurate results. However, scoring did not help to accurately identify the change in lesion dimensions, especially when the lesion size and changes were small; therefore, in this study, rather than categorizing periapical radiolucency based on the index parameter, periapical radiolucency was assessed based on the change in SPR (mm).

Using CBCT images, we used the largest linear distance(mm) as the SPR index. Most studies have evaluated changes in the volume of periapical lesion. Tsai et al. [26] measured the diameter of periapical lesions using CBCT, which showed high accuracy when the diameter was larger than 0.8 mm. In this study, one tooth had a SPR less than 0.8 mm in BO CBCT and three in AO CBCT. Most cases’ radiolucency were larger than 0.8 mm. Therefore, it is thought that evaluating SPRs using diameter (mm) is also a reliable method for evaluation, similar to evaluation based on volume.

This study has limitation of discrepancy in the sample selection process. Numbers of cases distributed into groups of patients’ sex, age, type of tooth and position of teeth were not balanced, and this might have influenced the study’s result. The future study with larger sample sizes and adequate number of cases distributed into each classification might provide more appropriate results.

## Conclusion

Despite the limitation in this study, orthodontic treatment had no significant impact on the size of periapical radiolucency in endodontically treated teeth. However, there was a significant difference in the changes in radiolucency size among females and among maxillary teeth. In both categories, radiolucency size decreased significantly.

## Data Availability

The datasets used and analyzed during the study are available from the corresponding author upon reasonable request.

## Abbreviations

SPR: Size of periapical radiolucency
ETT: Endodontically treated tooth
BO CBCT: CBCT images before orthodontic treatment
AO CBCT: CBCT images after orthodontic treatment

## References

[1] Vier FV, Figueiredo JA (2002). Prevalence of different periapical lesions associated with human teeth and their correlation with the presence and extension of apical external root resorption. Int Endod J.

[2] Seo BM, Miura M, Gronthos S, Bartold PM, Batouli S, Brahim J (2004). Investigation of multipotent postnatal stem cells from human periodontal ligament. Lancet.

[3] Linkhart TA, Mohan S, Baylink DJ (1996). Growth factors for bone growth and repair: IGF. TGF beta and BMP Bone.

[4] Brezniak N, Wasserstein A (2002). Orthodontically induced inflammatory root resorption. Part I: The basic science aspects. Angle Orthod.

[5] Li Y, Jacox LA, Little SH, Ko CC (2018). Orthodontic tooth movement: The biology and clinical implications. Kaohsiung J Med Sci.

[6] Alhadainy HA, Flores-Mir C, Abdel-Karim AH, Crossman J, El-Bialy T (2019). Orthodontic-induced external root resorption of endodontically treated teeth: A meta-analysis. J Endod.

[7] Mah R, Holland GR, Pehowich E (1996). Periapical changes after orthodontic movement of root-filled ferret canines. J Endod.

[8] Liang YH, Jiang L, Gao XJ, Shemesh H, Wesselink PR, Wu MK (2014). Detection and measurement of artificial periapical lesions by cone-beam computed tomography. Int Endod J.

[9] Tavares PB, Bonte E, Boukpessi T, Siqueira JF, Lasfargues JJ (2009). Prevalence of apical periodontitis in root canal-treated teeth from an urban French population: Influence of the quality of root canal fillings and coronal restorations. J Endod.

[10] Moreno JO, Alves FR, Gonçalves LS, Martinez AM, Rôças IN, Siqueira JF (2013). Periradicular status and quality of root canal fillings and coronal restorations in an urban Colombian population. J Endod.

[11] Heboyan A, Avetisyan A, Karobari MI, Marya A, Khurshid Z, Rokaya D (2022). Tooth root resorption: A review. Sci Prog.

[12] Amaro ERS, Ortiz FR, Dorneles LS, Santos MS, Barrioni BR, Miranda RM (2020). Estrogen protects dental roots from orthodontic-induced inflammatory resorption. Arch Oral Biol.

[13] Hashimoto M, Hotokezaka H, Sirisoontorn I, Nakano T, Arita K, Tanaka M (2013). The effect of bone morphometric changes on orthodontic tooth movement in an osteoporotic animal model. Angle Orthod.

[14] Macari S, Duffles LF, Queiroz-Junior CM, Madeira MF, Dias GJ, Teixeira MM (2015). Oestrogen regulates bone resorption and cytokine production in the maxillae of female mice. Arch Oral Biol.

[15] Hajihassani N, Ramezani M, Tofangchiha M, Bayereh F, Ranjbaran M, Zanza A, et al. Pattern of endodontic lesions of maxillary and mandibular posterior teeth: A cone-beam computed tomography study. J Imaging. 2022;8:290. 10.3390/jimaging8100290 PMC9605447 36286384

[16] Schaeffer MA, White RR, Walton RE (2005). Determining the optimal obturation length: A meta-analysis of literature. J Endod.

[17] Allison DA, Michelich RJ, Walton RE (1981). The influence of master cone adaptation on the quality of the apical seal. J Endod.

[18] Alqerban A, Almanea A, Alkanhal A, Aljarbou F, Almassen M, Fieuws S (2019). Impact of orthodontic treatment on the integrity of endodontically treated teeth. Eur J Orthod.

[19] Song M, Jung IY, Lee SJ, Lee CY, Kim E (2011). Prognostic factors for clinical outcomes in endodontic microsurgery: A retrospective study. J Endod.

[20] Barone C, Dao TT, Basrani BB, Wang N, Friedman S (2010). Treatment outcome in endodontics: The Toronto study–Phases 3, 4, and 5: Apical surgery. J Endod.

[21] Liao WC, Lee YL, Tsai YL, Lin HJ, Chang MC, Chang SF (2019). Outcome assessment of apical surgery: A study of 234 teeth. J Formos Med Assoc.

[22] Eckerbom M, Flygare L, Magnusson T (2007). A 20-year follow-up study of endodontic variables and apical status in a Swedish population. Int Endod J.

[23] Pallarés-Serrano A, Glera-Suarez P, Tarazona-Alvarez B, Peñarrocha-Diago M, Peñarrocha-Diago M, Peñarrocha-Oltra D (2021). Prognostic factors after endodontic microsurgery: A retrospective study of 111 cases with 5 to 9 years of follow-up. J Endod.

[24] Estrela C, Bueno MR, Azevedo BC, Azevedo JR, Pécora JD (2008). A new periapical index based on cone beam computed tomography. J Endod.

[25] Torabinejad M, Rice DD, Maktabi O, Oyoyo U, Abramovitch K (2018). Prevalence and size of periapical radiolucencies using cone-beam computed tomography in teeth without apparent intraoral radiographic lesions: A new periapical index with a clinical recommendation. J Endod.

[26] Tsai P, Torabinejad M, Rice D, Azevedo B (2012). Accuracy of cone-beam computed tomography and periapical radiography in detecting small periapical lesions. J Endod.

